# Should We Advance Our Understanding of Immunoglobulin E in Viral Immunity?

**DOI:** 10.1111/imm.70007

**Published:** 2025-06-13

**Authors:** Amanda Izeli Portilho, Valéria Oliveira Silva, Luis Fernando de Macedo Brigido, Elizabeth De Gaspari

**Affiliations:** ^1^ Immunology Center Adolfo Lutz Institute São Paulo São Paulo Brazil; ^2^ Post‐Graduate Program Interunits in Biotechnology University of São Paulo São Paulo São Paulo Brazil; ^3^ Virology Center Adolfo Lutz Institute São Paulo São Paulo Brazil; ^4^ Post Graduate Program Public Health Surveillance Disease Control Coordination São Paulo São Paulo Brazil

**Keywords:** antibody functionality, humoral response, IgE, immune response, viruses

## Abstract

Immunoglobulin E has been extensively studied in allergies and parasitic diseases. However, antigen‐specific IgE has been identified as part of the humoral response to some viruses, including Respiratory syncytial virus (RSV), Human rhinovirus (HRV), Influenza, Hepatitis B virus (HBV), Human immunodeficiency virus (HIV), Herpes simplex virus (HSV), Dengue virus (DENV) and SARS‐CoV‐2. In this brief article, we have reviewed key aspects of IgE function and structure, and summarised the findings about this antibody in virus‐specific immune response. To date, IgE effector mechanisms in the face of viruses have been almost unexplored through functional assays. We speculate on possible functionalities, such as neutralisation, cytotoxicity and immunopathology of viral diseases, and provide insights about gaps to fill in future research.

## Graphical abstract

Immunoglobulin E has been extensively studied in allergies and parasitic diseases. Antigen‐specific IgE has been identified as part of the humoral response to viruses. This revision speculates on possible functionalities for IgE, such as neutralisation, cytotoxicity and immunopathology of viral diseases, and provide insights about gaps to fill in future research.

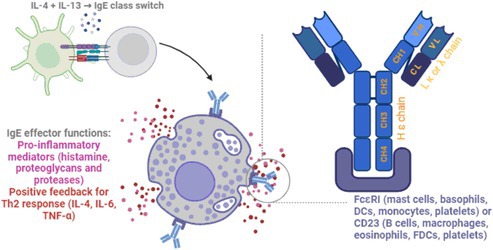

## 1 Introduction

Viruses are non‐living organisms that require a host cell to replicate. They consist of their genetic core (DNA or RNA), enclosed by a protein layer (capsid), and, in some cases, present a lipid envelope [1]. They are responsible for many infections, posing a significant burden on human health. According to the World Health Organisation (WHO), several diseases listed as leading causes of death are associated with viruses—for instance, many diarrheal, respiratory and systemic diseases are associated with enteroviruses, Influenza and HIV, respectively [2]. Additionally, COVID‐19 has been a major global health concern, accounting for over 7 million deaths worldwide since its emergence 5 years ago [3]. Although pathogenic viruses vary according to environmental stability, replication strategies, transmission route, tissue tropism and other characteristics, understanding their functionality and host interactions is fundamental for combating infectious diseases, developing vaccines and advancing medical research [4].

Antibodies, or immunoglobulins (Ig), are proteins secreted by antibody‐secreting cells (ASCs), which differentiate from activated B lymphocytes. Antibodies present Fc and Fab regions; while the Fc interacts with cell receptors, mediating biological functions, the Fab region binds (ideally) to non‐self, harmful structures, such as pathogen epitopes [5, 6]. Humans secrete different antibody classes (IgG, IgM, IgA and IgE), and also express IgD, which functions as a cell receptor and, in secreted form, contributes to the homeostasis of the humoral response and immune surveillance at mucosal sites, especially in the nasopharynx, as reviewed before [7, 8].

The IgE response is more commonly studied in parasitic diseases and allergies, as these antigens are known to trigger this antibody class [9, 10, 11, 12]. However, evidence suggests that IgE also play a role in different contexts, binding specifically to antigens from bacteria, as *Haemophilus influenzae* , *Streptococcus pneumoniae* , *S. pyogenes*, *Brucella* spp., *E. coli* and *Staphylococcus aureus* [13, 14, 15, 16, 17, 18, 19]; as well as respiratory viruses, including Respiratory syncytial virus (RSV), Human rhinovirus (HRV), Influenza and SARS‐CoV‐2 [20, 21, 22, 23, 24, 25, 26] and viral systemic diseases, such as Hepatitis B virus (HBV), Human immunodeficiency virus (HIV), Herpes simplex 1 (HSV‐1) and Dengue virus (DENV) [27, 28, 29, 30, 31].

Despite that, little is known about the role of IgE in such contexts. It is reasonable to expect that this immunoglobulin could act through different mechanisms. Antibodies' contribution to viral protection includes: direct neutralisation of the pathogen, antibody‐dependent cytotoxicity (ADCC), antibody‐dependent cellular phagocytosis (ADCP) and antibody‐dependent complement deposition (ADCD) [32], as summarised in Table 1. The last column shows current evidence for IgE non‐canonical roles usually attributed to other Ig classes.

**TABLE 1:** Mechanisms by which antibodies fight viral infections and evidence for IgE functionality.

Function	Immune mechanism	Evidence for IgE functionality (References)
Neutralisation	Inhibition of virus‐receptor binding and, consequently, internalisation and replication	A statistical correlation was observed between anti‐RBD IgE and SARS‐CoV‐2 neutralisation, the same was not observed for the DENV [23, 27]. Purified IgE should be tested to prove virus neutralisation capacity, as investigated for HIV‐1. In this case, the in vitro infection of cells suggests a lack of neutralisation [30]
Cell degranulation	Release of granule content (chemical mediators, enzymes and other molecules), resulting in pro‐inflammatory signalling	Basophils of patients expressing HSV‐1‐specific IgE were activated after virus‐lysate stimuli in vitro. Positive‐IgE sera and HSV‐1 incubation also resulted in degranulation of a basophil cell lineage [28]
Antibody‐dependent cell cytotoxicity (ADCC)	Release of cytotoxic enzymes that promote lysis of the infected cell	IgE‐mediated ADCC was observed in pancreatic adenocarcinoma and ovarian carcinoma, and possibly controlled HIV‐1 production [30, 33, 34]
Antibody‐dependent cellular phagocytosis (ADCP)	Phagocytic cells engulf opsonized targets, limiting their spread and facilitating antigen presentation	A monoclonal IgE anti‐ovarian tumour induced ADCP of monocytes upon IL‐4 stimuli and CD23 signalling. No viral IgE‐ADCP was described so far [34]
Antibody‐dependent complement deposition (ADCD)	Ig binds to the target and to Cq1, initiating the classical complement pathway, which induces inflammation and membrane‐attack complex	None described so far

Abbreviations: DENV, Dengue virus; HIV‐1, Human immunodeficiency virus‐1; HSV‐1, Herpes simplex virus‐1; SARS‐CoV‐2, Severe acute respiratory syndrome coronavirus 2.

It is important to emphasise that, although studies suggested that IgE may play a role in immunity against certain viruses, its exact function remains unclear. For example, IgE was associated with the immune response against SARS‐CoV‐2 [21, 22, 24], yet its significance in antiviral response appears to be less prominent than other types of antibodies, such as IgG or IgA [35, 36]. Considering these findings, this brief review intends to summarise the current knowledge of IgE response to viruses and speculate its role on immunity.

## 2 Biology and Function of Human IgE

IgE was the last antibody class to be discovered. It was recognised in the 1960s as a distinct immunoglobulin from the previously known IgA, IgD, IgG and IgM, and identified as a key player in the mechanisms of immediate hypersensitivity [37]. It has a central role in the immune response to allergens, being responsible for activating mast cells and basophils, and its discovery contributed immensely to allergy treatments [38, 39]. In addition, its role in eosinophil activation has been studied in the parasitology field, improving the understanding of the immune response to helminths and leading immunologists to discuss the interplay between infectious diseases and allergies [40, 41].

The IgE immunoglobulin is a monomer composed of two ε‐heavy and two light (κ or λ) chains. Its C region presents four constant (Cε1—4) and one variable domains, allowing interaction with two cell receptors, FcεRI and FcεRII (or CD23). Different from IgG and IgA monomers, IgE does not have a hinge region; however, the immunoglobulin may adopt an open or closed conformation, which dictates the binding specificity: while the first form binds to FcεRI, the latter binds to CD23 [42, 43]. The half‐life of circulating IgE is approximately 1 day. Noteworthy, the scenario changes when this molecule is bound to its receptor, thus persisting through weeks, resulting from a high affinity interaction [38, 44].

FcεRI is the high‐affinity receptor for IgE, expressed by mast cells, basophils, dendritic cells (DCs), monocytes, platelets, and neutrophils of asthmatic patients [45, 46, 47]. Its expression is upregulated by IgE, which also promote mast cell survival and expansion [38, 42]. On the other hand, CD23 is the low‐affinity receptor for IgE, found in B cells, activated macrophages, eosinophils, follicular DCs and platelets [46, 47]. The glycosylation of IgE antibodies by N‐glycans affect the receptor binding and overall effector response [48].

As described in the literature, the primary effector function of IgE is antigen binding—typically to an allergen or parasite—promoting the degranulation of eosinophils, basophils and/or mast cells. In the allergic context, IgE binding to the mast cells and basophils leads to the accumulation of cytosolic calcium, prostaglandin and leukotriene synthesis, resulting in the secretion of hypersensitivity mediators, such as histamine, proteoglycans and proteases, which mediate vasodilation, tissue edema, mucus production and smooth muscle constriction; and enhanced secretion of IL‐4, TNF and IL‐6 cytokines [39, 49]. Meanwhile, eosinophils activated by IgE during parasitic infections degranulate, releasing several proteins with anti‐helminthic properties, lipid mediators and pro‐inflammatory molecules [50].

**FIGURE 1:**
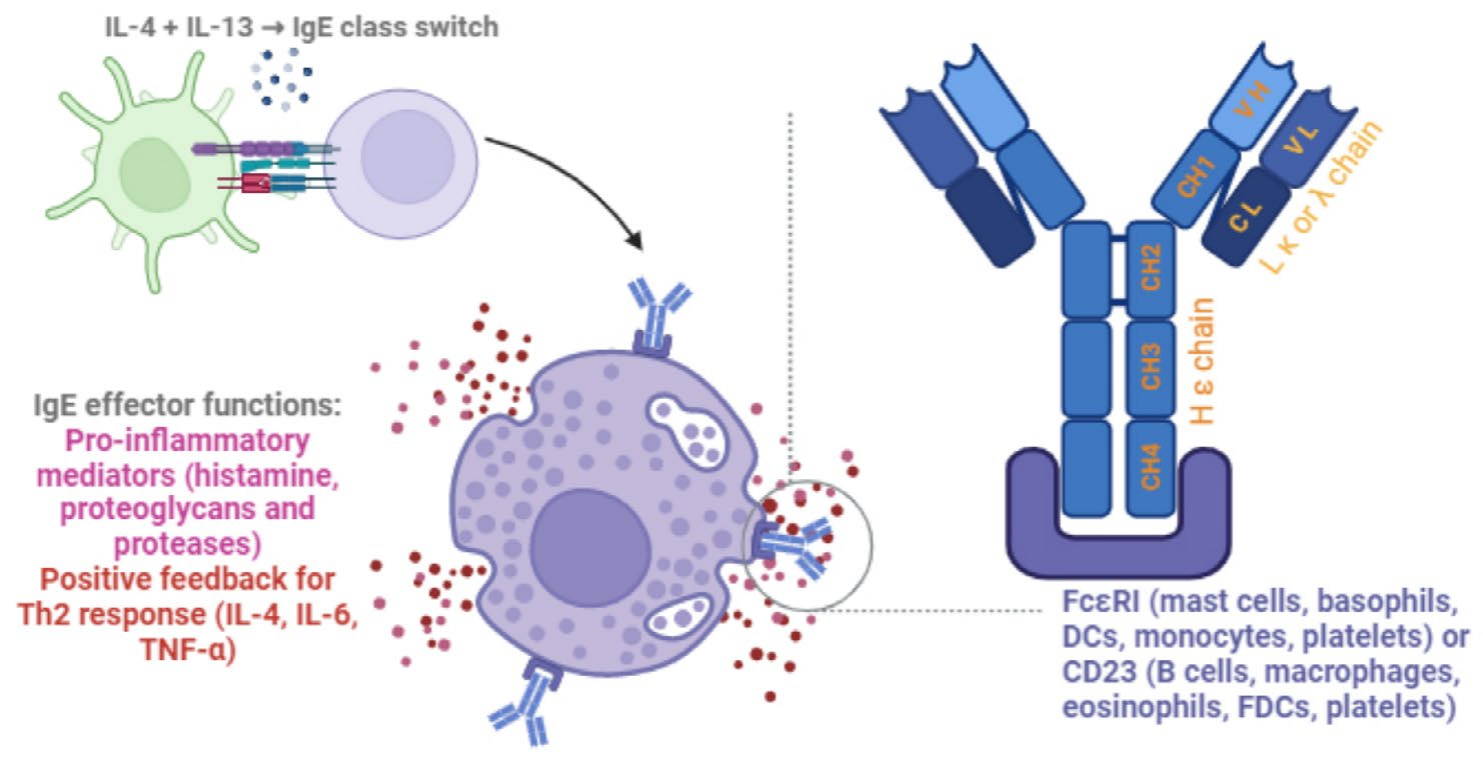
Summary of the main events to propagate IgE class‐switch, its structure and effector functions. Strong IL‐4 and IL‐13 signalling promotes IgE class‐switch. Once secreted, the immunoglobulin binds to the antigen and activates the cellular response of cells expressing FcεRI or CD23 to secrete pro‐inflammatory molecules and positive feedback to Th2 response. (Figure created with BioRender).

High IgE levels are considered a hallmark of Th2 responses, as B cells require strong IL‐4 and IL‐13 signalling to undergo class‐switch for IgE [51]. Even though this antibody class is analysed in allergies (as an undesired, often deleterious immune response), there are some hypotheses to explain why IgE could confer evolutionary advantage. First, IgE and Th2 responses may support tissue repair after parasitic infections, as IL‐4 signalling decreases IL‐17 and enhances IL‐10 secretion [52]. In a more direct role, IgE can be perceived as a sensor, either for toxins or other antigens, triggering a localised immune response that prevents or minimises systemic dissemination as well as mechanical responses that may be protective, such as itching, triggering the urge to scratch. Regarding toxins, IgE could bind the molecules even at low concentrations, facilitating neutralisation and signalling an immune response. As a surveillance to diverse antigens, B cells expressing CD23 could uptake IgE‐coupled antigens, thus augmenting IgG production [53, 54]. It has been postulated for many years that IgE‐secreting plasma cells were short‐lived, but increasing evidence shows some cells may also be long‐lived, suggesting a role in chronic diseases [51]. In addition, two investigations recently described memory B cells expressing IgG1, CD23 and IL‐4Rα in patients with food allergy or rhinitis, which were likely to be a source for long‐lived IgE‐secreting cells [55, 56]. Figure 1 illustrates IgE structure and summarises its known functions.

IgE concentration in serum is very low—the general hierarchy of systemic antibodies is IgG > IgM > IgA > IgE—IgD, the exception occurring in mucosal compartments, where IgA is the dominant class. This pattern is maintained even when specific immune responses to common pathogens are considered, as bacteria and viruses. Due to its overall low concentration, studying IgE often requires assays with enhanced sensitivity and appropriate controls [6, 8, 57].

## 3 Respiratory Viruses Induce Specific‐IgE

Studies describing IgE specific to viral antigens have been published since the 80s, mostly focused on respiratory viruses. Respiratory‐Syncytial‐Virus (RSV) is one of the main causes of severe respiratory infection in infants, and until recently, no vaccines were available [58]. IgE‐specific antibodies were detected in RSV‐infected individuals for many years [20, 59, 60, 61]. Different paediatric studies have reported an association between RSV‐specific IgE and clinical symptoms, such as wheezing and more aggravated/severe disease, including involvement of the lower respiratory tract system, like pneumonia [20, 60, 62]. Interestingly, not only IgE, but also histamine levels were increased in the nasopharyngeal secretion of wheezing patients [61]. The same group described that individuals who suffered from bronchiolitis had IgE bound to epithelial cells in the mucosa airway [63] and, assessing the ratio between nasopharyngeal and seric IgE, they found that mucosal IgE was twice as high. However, no causal relationship was established from these studies, which revealed that other factors, such as atopy history and passive smoking, influenced patient outcomes too.

Adding to the complexity of the topic, another group detected RSV‐specific IgE in only one patient out of an 81‐children cohort, despite optimising the capture‐ELISA assay for increased sensitivity [64]. Thus, the authors had limited information about their cohort, so it would not be possible to properly compare the studies and speculate a hypothesis for the divergent results.

More recently, anti‐Influenza A IgE was found in a small cohort up to 20 months after vaccination—among the six individuals studied, five had detectable IgE, and only two were atopic [25]. Another study involving 44 children found that patients diagnosed with Influenza Vaccine Atopy (IVA) had significantly higher levels of IgE specific to the hemagglutinin antigen from different vaccine manufacturers, but vaccine excipients, like formaldehyde and thimerosal, were not recognised by IgE antibodies [65].

Different research groups have documented the presence of IgE following natural infection with SARS‐CoV‐2 through comprehensive serological analysis. In the cohort of Plüme et al. [22], IgE recognised Spike and Nucleocapsid proteins, some patients maintaining detectable levels up to 86 days post‐infection, and IgE levels showed a positive correlation with disease severity, with N‐specific IgE distinguishing mild and moderate/severe patients. Gimenez‐Orenga et al. [21] studied 22 acute COVID‐19 cases and found IgE mainly to the Nucleocapsid protein, whereas 12 long‐COVID patients had IgE directed to the Spike. IgE levels exhibited a Spearman correlation of 0.8 with post‐COVID‐19 symptoms. However, it is important to emphasise that the study was limited by a small sample size. Given that IgE positivity was particularly high in more severe COVID‐19 cases, both authors suggested that mast cell activation could contribute to exacerbated inflammation during the infection. Our group investigated IgE responses following SARS‐CoV‐2 natural infection and vaccination. As our cohort included only asymptomatic and mild COVID‐19 cases, we did not observe any correlation between IgE levels and disease severity, and no specific symptoms were associated with increased IgE. Considering vaccines, we compared the response to inactivated and viral‐vector vaccines boosted by mRNA. IgE levels were increased in viral‐vector vaccines, but after the mRNA booster, no differences were observed. These results led our group to suggest that IgE kinetics would follow a similar response to IgG, although no particular roles were verified for this antibody [23]. More recently, de la Poza [24] studied COVID‐19 patients and found a similar prevalence of IgE antibodies across mild, severe and critical presentations. Thus, among ferritin, D‐dimer, C‐reactive protein (CRP) and lactate dehydrogenase (LDH), only LDH had an association with IgE, which provides evidence against the hypothesis that IgE contributes to COVID‐19 inflammation.

## 4 What Triggers Virus‐Specific IgE?

As mentioned in the previous section, respiratory viruses (and even bacteria) triggered specific IgE. A plausible link between IgE and upper‐respiratory infections is atopy, mainly represented by asthma. This is a chronic condition characterised by exacerbated inflammation and obstruction of the airways, and its severity differs among patients. The disease is immune‐mediated and several cell types and cytokine pathways are involved, especially T cells, eosinophils, mast cells, and neutrophils, with pro‐inflammatory and type 2 signalling, all activated upon allergen encounter [66, 67].

Asthma is a multifactorial condition, where environmental and patient characteristics affect disease development, from air pollution, occupational exposure to common allergens (pollen, insects, seafood, etc.), to smoking, stress and obesity [68]. The individual's genetics may predispose Th2 responses, resulting in higher chances of developing atopies. SNPs of genes from several chromosomes were pointed out as asthma predictors, these relate to lung function, IL‐1 (pro‐inflammatory cytokine) and IL‐33 (activates Th2, innate lymphoid cells type 2 and IgE synthesis) [69]. The genetics of asthma pathophysiology has been reviewed recently [70].

In addition to genome‐based approaches, epigenetic mechanisms have been suggested, and noncoding RNAs seem to contribute to asthma susceptibility as well. Differently methylated CpG regions were found in immune, lung and airway cells of children hospitalised with bronchiolitis, which were associated with IgE levels and lung function [71]. microRNAs (miRNA) related to neutrophils and T helper cells were differently expressed in the nasal samples of hospitalised infants with severe bronchiolitis. Moreover, these miRNAs seemed to affect FcεR signalling, suggesting an explanation for the increased risk of developing asthma when there is a history of bronchiolitis [72]. Likewise, long noncoding RNAs were associated with IgE sensitisation, FcεR signalling and Th2 cytokines, such as IL‐4 and IL‐13 [73]. Together, these studies raise different molecular mechanisms where RNA could be a player in the pathophysiology of bronchiolitis and the development of asthma. The role of microbiome was less studied so far, but a comprehensive analysis of 450 hospitalised infants characterised the patient's nasopharyngeal airway metagenome, and found the group who had a high abundance of rhinoviruses A and C, and *H. influenzae* to have a higher risk of developing asthma [74].

Indeed, many investigations of virus‐specific IgE were conducted in atopic patients presenting with asthma [26, 31, 63], atopic dermatitis [28], previous history of allergies [25, 26, 75]; or at least showing characteristics associated with it, such as bronchiolitis [20, 59, 60, 61], and familial history [20, 60]. Suggesting that individual predisposition for allergic responses influences virus‐specific IgE agrees with scientific evidence. However, this could not be the only factor, given that some of the studies cited above showed specific IgE in healthy patients without atopic disease [25, 26, 31, 75]. Likewise, B cells characterised by a type 2 memory phenotype specific for SARS‐CoV‐2 were present in non‐allergic and allergic patients [55]. Other studies, conducted in non‐atopic cohorts, found IgE binding to viral antigens as well [21, 22, 23, 24, 27, 29]. Moreover, it is not clear if viral infections trigger asthma or if the patient's predisposition for atopic diseases renders them susceptible to respiratory infections; much less how all that supports the IgE response to viruses.

Studies in mouse models suggested that viral infection skewed the immune response to the Th2 pattern and IgE production. Neonatal, wild‐type (WT) mice nasally infected with RSV developed an IgE response and, when reinfected, they presented airway hyperresponsiveness (AHR) with eosinophilia, mucous production, and IL‐4, IL‐5 and IL‐13 secretion. These AHR effects were attenuated in IL‐4^−/−^/IL‐13^−/−^ and FcεR^−/−^ knockout mice. Interestingly, in the same study, the authors performed a sensitisation experiment, treating WT mice with RSV‐IgE or ovoalbumin (OVA)‐IgE. Upon RSV challenge, mice sensitised with RSV‐IgE developed AHR, but the same was not observed with OVA‐IgE. Treating mice with anti‐IgE decreased the AHR effects [76]. Another study performed OVA sensitisation with or without previous infection with Influenza A virus. The infected group had elevated anti‐OVA IgE and an increase in T cell counts in the lungs, especially CD8+, when compared with the group sensitised without viral infection. The authors discussed their results with interesting studies. They suggested that, once respiratory viral infections diminish macrophages (responsible for antigen clearance) and lead to phenotypic changes in CD8+ cells to co‐express Th1 and Th2 cytokines, this environment could promote IgE production [77].

Grayson and collaborators also addressed this issue. First, to understand how a typical Th1 antiviral response is surpassed by a Th2 polarisation, they used a mouse model infected with Sendai virus (the murine parainfluenza virus). After infection, lung DCs started expressing FcεRα (and continued until 2–3 weeks post‐infection), an effect mediated by the increase in type I IFN. Thus, cross‐linking of FcεR resulted in DCs secreting CCL28, a chemoattractant for CD4+ cells, which produced the IL‐13, a cytokine required for a strong Th2 response, which mediates cell metaplasia and atopic disease [78]. Thus, the group assessed how their findings would translate from the mouse model to humans. They isolated DCs from the PBMC of atopic and non‐atopic volunteers. DCs from both groups expressed IgE, although it was higher in the atopics. Culturing DCs with a cross‐linking of FcεR resulted in enhanced CCL28, especially in the non‐atopic group. The authors discussed that this intriguing result could be due to elevated IgE in DCs from atopic individuals, preventing the binding of the cross‐linker. Despite that, they confirmed that human DCs secrete the same chemoattractant for CD4+ [79].

This topic gets even more interesting when different viruses are investigated. For example, during the COVID‐19 pandemic, the burden of SARS‐CoV‐2 was not more aggravated in atopic than non‐atopic patients. An experimental study attributed this effect to IL‐13 reducing ACE‐2 expression by epithelial cells [80]. Such different results highlight that each infection must be considered according to its unique characteristics.

From a different perspective, it could be argued that viral vaccines contribute to IgE induction due to aluminium hydroxide, an adjuvant known to polarise Th2 responses. This predominance of Th2 responses to alum was assessed by several studies. For example, human peripheral blood mononuclear cells (PBMC) presented enhanced expression of IL‐4 upon aluminium hydroxide exposure, which enhanced MHC‐II expression, favouring antigen presentation [81]. Experimental studies using protein antigens from either parasitic, bacterial, or viral pathogens observed a strong Th2 profile as well, with predominance of mouse‐IgG1 isotype, IL‐4 and IL‐5 secretion [82, 83]. Interestingly, a study found that this adjuvant could polarise Th2 response even when IL‐4 and IL‐13 (two classic type 2 cytokines) were blocked, suggesting other underlying mechanisms [84].

Recombivax, for Hepatitis B virus, and CoronaVac, for SARS‐CoV‐2, contain alum, but FluMist and Fluzone, for Influenza, ChAdOx1 and BNT162b2, for SARS‐CoV‐2, do not [23, 25, 31, 85]. Moreover, if we consider only SARS‐CoV‐2 vaccines, ChAdOx1 and BNT162b2, without alum, resulted in even more robust IgE levels than CoronaVac, which uses this adjuvant, suggesting that it was not even an IgE enhancer [23]. Thus, natural infections resulted in antigen‐specific IgE in the absence of adjuvants [21, 22, 24, 28, 29, 75].

Supporting the role of viral structures in inducing antigen‐specific IgE, Ramos cell lineage (derived from human Burkitt lymphoma) and B cells (isolated from whole blood) infected in vitro with Measles, Mumps, Rubella (MMR) vaccine exhibited IgE class‐switching [85]. The same group described that a protein kinase activated by double‐strand RNA (a common viral PAMP) mediated IgE class switch of Ramos cells identified by transcription of ε‐chain; thus, infecting these cells with rhinovirus yielded similar results [86].

Smith‐Norowitz and collaborators documented IgE antibodies against Hepatitis B surface antigen (HBs) in a cohort of 33 asthmatic and non‐asthmatic children vaccinated with Hepatitis B subunit vaccine, where both groups presented IgE‐anti‐HBs [31]; in addition, Pellegrino et al. reported IgE‐anti‐HIV‐1 in children who acquired HIV through maternal transmission [30]. A cohort of 168 patients infected with DENV also had virus‐specific IgE, with elevated levels in those who developed hemorrhagic fever and/or shock syndrome [29]. A study with atopic dermatitis patients showed specific IgE for HSV‐1, a primarily mucosal infection, which can spread systemically. The authors also demonstrated that patients' IgE activated basophils [28]. These findings pertain to systemic diseases, caused by viruses transmitted via parenteral, sexual or vector routes, suggesting that the pathogen itself, rather than the infection route, plays a role in triggering IgE production.

## 5 Potential Roles for IgE in Viral Infection and Immunity

A relationship between respiratory infections and atopic conditions, like asthma, has been observed, which suggests that antivirus IgE is more relevant in the respiratory environment. Indeed, IgE was found in the nasal fluids of children infected with Parainfluenza virus, with elevated levels in those who presented with croup and wheezing symptomatology, and the authors suggested that these antibodies might play a role in disease pathogenesis [87]. When RSV infection was studied, specific IgE levels in the nasopharyngeal secretions correlated with antigen load and pneumonia [59]. Beyond exacerbating respiratory diseases, such as asthma, IgE may also exacerbate systemic inflammatory response, which is a serious issue for viral infections. Plume et al. [22] suggested that IgE could contribute to inflammation in COVID‐19, provided that other authors reported mast cell activation during the infection and, in their work, IgE was mainly observed in severe cases. Similarly, increased IgE and histamine levels were observed in RSV‐infected patients with wheezing, correlating with hypoxia [61]. From a systemic perspective, enhanced IgE levels were detected in COVID‐19 patients with reduced physical functioning post‐infection, suggesting a worsened disease outcome [21], and higher anti‐DENV IgE titers have been suggested as a predictor for poor prognosis in cases with systemic presentation, such as hemorrhagic fever and shock syndrome [29]. As in vitro evidence, Influenza A acted as a histamine release enhancer in leukocytes isolated from septic donors through an IgE‐dependent mechanism [88]. Taken together, these findings suggest that a virus‐specific IgE response may contribute to inflammation and, although important for immune activation, could lead to systemic complications, proving detrimental to the patient. Nonetheless, contradictory studies may be found. An investigation with DCs from asthmatic cohorts observed that high IgE levels and signalling through FcεRI reduced secretion of the pro‐inflammatory type I interferons (IFN) in response to Influenza A and rhinovirus [89, 90].

On the other hand, IgE can trigger cytotoxic effect via Fcε Receptors and could have neutralising properties, as it tends to bind antigens with great avidity [49, 52]. If inflammation remains controlled, such effects could be beneficial. However, no studies to date have tested purified IgE for direct neutralisation. In our SARS‐CoV‐2 vaccinees cohort, IgE titers and avidity correlated with neutralisation [23], but results from a dengue‐infected cohort suggested otherwise, with anti‐DENV IgE showing no correlation with neutralisation [27]. An in vitro experiment infecting human PBMC with HIV‐1 virus found that the addition of sera samples positive for HIV‐IgE before the infection did not inhibit viral replication, further suggesting a lack of neutralising effect of this antibody [30].

Other studies suggested that IgE may mediate cytotoxic effect, potentially acting against viral and neoplastic diseases [91]. In vitro studies with ovarian cancer cells demonstrated that MOv18, a monoclonal IgE developed for treating this neoplasia, mediated ADCC via FcεRI, and ADCP via CD23 and IL‐4 stimuli in monocytes. To confirm the results, the group treated a mouse model transplanted with human ovarian carcinoma xenograft, and observed the best survival outcomes when human PMBCs were administered with MOv18. Interestingly, the same article described an eosinophil‐mediated cytotoxicity to cancerous cells [34]. A similar effect was observed for 12 pancreatic cancer patients. Total IgE and soluble CD23, an IgE receptor, were elevated in patients compared with healthy controls, but no differences were recorded for other immunoglobulin classes. When Human pancreatic adenocarcinoma cell lines (HPAC) were incubated with patient sera, IgG and IgE binding were detected. In HPAC co‐culture with PBMC, cytotoxicity was observed when patient sera and purified IgE were mixed, but decreased if anti‐human IgE was added, confirming a contribution of this Ig class for ADCC [33].

The potential of IgE to induce ADCC and ADCP is being explored in therapeutic applications, particularly through monoclonal antibodies (mAbs). An anti‐CD38 IgE demonstrated promising results as a treatment for multiple myeloma, showing efficacy both in vitro and in vivo in BALB/c mice expressing FcεRI. Additionally, it prolonged survival in a xenograft mouse model [92], and an engineered IgE with the antigenic specificity of Trastuzumab, a humanised IgG1 used to treat breast cancer, was shown to be effective by in vitro studies [93]. Moreover, studies have suggested that individuals with IgE deficiency may have an increased risk of developing neoplastic diseases, reinforcing the effector role of this antibody beyond allergy and autoimmunity [94]. Unfortunately, we could not find similar studies for antiviral therapies.

From infectious diseases, one paper suggested that HIV‐IgE contributed to controlling the infection. In this study, 11 children had IgE specific to the virus and no detectable antigenemia. In vitro infection of PBMCs with HIV‐1 was reduced by 10% if sera from these patients were added before the infection, whereas a 95% reduction was observed when sera were added after infection, leading the authors to suggest that IgE did not neutralise the virus directly, but controlled replication through other mechanisms, likely to be ADCC. These findings were further confirmed by depleting IgE from sera and adding anti‐human IgE to the culture, which reversed the results [30]. In the investigation of an atopic cohort infected by HSV‐1, the authors demonstrated IgE‐mediated activation of basophils in two distinct experiments. First, they analysed blood samples from the patients after stimuli with HSV‐1 lysate, observing activation in relation to total basophils. After, they performed passive sensitisation of a basophil cell lineage with the patient's serum containing specific IgE, then incubated the cells with HSV‐1 and quantified β‐hexosaminidase in the culture supernatant, which showed basophil degranulation [28].

Apart from effector functions in viral immune response, multiple roles for IgE are being discussed. As recently reviewed, this antibody class could act on antigen presentation, boosting T and B cells' response and others. Elucidating FcεRI and CD23 interactions with cell populations and the cytokine effects on different isoforms of the same receptor are warranted [95].

## 6 Conclusion and Perspectives

Specific IgE responses have been documented for many non‐parasitic infections, especially viruses, involving different ecological niches, transmission routes, and tissue tropism within the host. As observed in this review, virus‐specific IgE has been described since the 1980s, but little is known about its biological functionality. There is evidence that this antibody mediates immune functions via ADCC and ADCP, mainly corroborated by studies from another area (neoplastic diseases), and controversial data about virus neutralisation. Studies assessing the functionality of IgE antibodies in viral diseases would help elucidate other roles IgE might have. This gap could be addressed by methodologies as virus neutralisation, for which there are several protocols, such as virus neutralisation test (VNT), surrogate‐ELISA (sVNT), pseudovirus neutralisation assay, and plaque reduction neutralisation test (PRNT); and opsonophagocytic and cytotoxicity assays to assess ADCP and ADCC. These are well‐standardised for flow cytometry, but novel luminescence assays are being proposed [96, 97]. It is important to highlight that serum samples are dominated by IgG, which competes with IgE, probably interfering with the results. Protocols to deplete samples from IgG, using protein A/protein G, for example, could be explored; and IgE purification would provide even more reliable results [98]. Animal models would be important for performing controlled mechanistic studies. Finally, revisiting previous studies, testing stored samples or analysing data, and observing associations between symptomatology, atopy history, and virus‐immune response could provide insights for future research.

This review suggests that IgE could have unknown or complex functions in viral infections, potentially paving the way for novel therapeutic strategies. IgE mAbs are being explored to enhance ADCC in cancer treatment, based on characteristics as the stability of its high‐affinity interaction with cell receptor, capacity to infiltrate solid tumours, and lack of intrinsic FcεR inhibitors [43]. It is desirable that mAbs for antiviral therapies present high affinity for the pathogen, a characteristic that could be observed for IgE, as this antibody class requires prolonged exposure to the antigen to favour class‐switch [99]. However, the cellular functions of IgE should be well‐characterised to understand its systemic impact in the patient. This gap requires basic immunological studies to aid (or not) the development of IgE mAbs.

All that considered, the IgE‐response to viruses highlights a new opportunity in immunology for more extensive research in an under‐represented area, which could open up opportunities for new diagnostic/prognostic tools and treatments.

## Author Contributions

**Amanda Izeli Portilho:** conceptualization, investigation, writing – original draft. **Valéria Oliveira Silva:** conceptualization, investigation, writing – original draft. **Luis Fernando de Macedo Brigido:** conceptualization, investigation, supervision, writing – editing and revision. **Elizabeth De Gaspari:** conceptualization, investigation, supervision, writing – editing and revision.

## Conflicts of Interest

The authors declare no conflicts of interest.

## Data Availability

Data sharing is not applicable to this article as no new data were created or analyzed in this study.
